# MicroRNA-200c increases radiosensitivity of human cancer cells with activated EGFR-associated signaling

**DOI:** 10.18632/oncotarget.18924

**Published:** 2017-07-03

**Authors:** Taeryool Koo, Bong Jun Cho, Dan Hyo Kim, Ji Min Park, Eun Jung Choi, Hans H. Kim, David J Lee, In Ah Kim

**Affiliations:** ^1^ Department of Radiation Oncology, Graduate School of Medicine, Seoul National University, Seoul, Republic of Korea; ^2^ Medical Science Research Institute, Seoul National University Bundang Hospital, Seongnam, Republic of Korea; ^3^ Department of Radiation Oncology, Seoul National University College of Medicine, Seoul, Republic of Korea; ^4^ Cancer Research Institute, Seoul National University College of Medicine, Seoul, Republic of Korea

**Keywords:** microRNA-200c, EGFR-associated signaling network, radiosensitization, human cancer cells

## Abstract

MicroRNA-200c (miR-200c) recently was found to have tumor-suppressive properties by inhibiting the epithelial-mesenchymal transition (EMT) in several cancers. miR-200c also interacts with various cellular signaling molecules and regulates many important signaling pathways. In this study, we investigated the radiosensitizing effect of miR-200c and its mechanism in a panel of human cancer cell lines. Malignant glioma (U251, T98G), breast cancer (MDA-MB-468), and lung carcinoma (A549) cells were transfected with control pre-microRNA, pre-miR-200c, or anti-miR-200c. Then, RT-PCR, clonogenic assays, immunoblotting, and immunocytochemisty were performed. To predict the potential targets of miR-200c, microRNA databases were used for bioinformatics analysis. Ectopic overexpression of miR-200c downregulated p-EGFR and p-AKT and increased the radiosensitivity of U251, T98G, A549, and MDA-MB-468 cells. In contrast, miR-200c inhibition upregulated p-EGFR and p-AKT, and decreased radiation-induced cell killing. miR-200c led to persistent γH2AX focus formation and downregulated pDNA-PKc expression. Autophagy and apoptosis were major modes of cell death. Bioinformatics analysis predicted that miR-200c may be associated with *EGFR, AKT2, MAPK1, VEGFA*, and *HIF1AN*. We also confirmed that miR-200c downregulated the expression of VEGF, HIF-1α, and MMP2 in U251 and A549 cells. In these cells, overexpressing miR-200c inhibited invasion, migration, and vascular tube formation. These phenotypic changes were associated with E-cadherin and EphA2 downregulation and N-cadherin upregulation. miR-200c showed no observable cytotoxic effect on normal human fibroblasts and astrocytes. Taken together, our data suggest that miR-200c is an attractive target for improving the efficacy of radiotherapy via a unique modulation of the complex regulatory network controlling cancer pro-survival signaling and EMT.

## INTRODUCTION

MicroRNAs (miRNAs) are important regulators of cell signaling pathways crucial for the growth of human cancer cells [1]. Cancer-associated miRNAs are located downstream of major oncogenes and tumor suppressor genes that act as transcription factors [2]. Alterations in miRNAs can result in cancer genesis and progression. For example, levels of some miRNAs are decreased in human cancers [3]. Therefore, understanding the regulatory function of miRNAs during tumor progression will contribute to the development of targeted molecular therapies.

A member of the miRNA-200 family, miRNA-200c (miR-200c), recently was found to have tumor-suppressive properties by inhibiting the epithelial-mesenchymal transition (EMT) process in several cancers. In primary glioblastoma multiforme (GBM) tissues, miR-200c and E-cadherin were found to be downregulated when epidermal growth factor receptor (EGFR) was highly amplified [4]. *EGFR* wild-type non-small cell lung cancer (NSCLC) cell lines regained sensitivity to EGFR tyrosine kinase inhibitors when EMT was inhibited by miR-200c overexpression [5]. miR-200c also interacts with various cellular signaling molecules and regulates many important signaling pathways, such as STAT3, PI3K/Akt [6], and ERK [7]. Clinically, analysis of patient data using The Cancer Genome Atlas (TCGA) datasets showed that decreased miR-200 family expression was associated with poor overall survival in ovarian, renal, lung, and basal-like breast cancers [8]. However, at this time, it is not clear whether miR-200c has a radiosensitizing effect in human cancer cells.

In the present study, we investigated the radiosensitizing effect of miR-200c and the mechanism of radiosensitization in a panel of human cancer cell lines with activated EGFR-associated signaling.

## RESULTS

### Ectopic overexpression of miR-200c increases the radiosensitivity of human cancer cells with activated EGFR signaling

**Figure 1:**
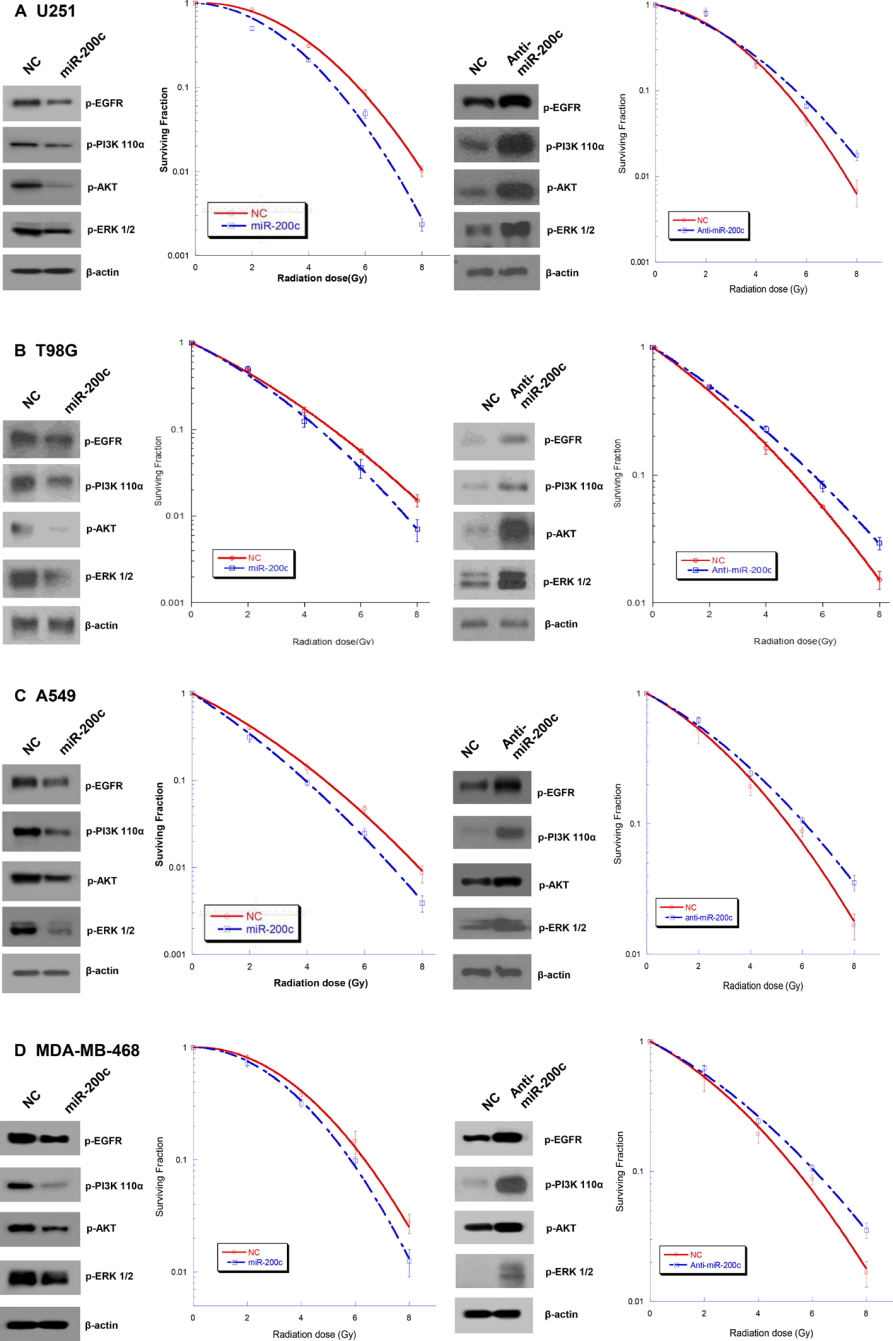
Effects of miR-200c on radiation response and EGFR-associated signaling Ectopic overexpression of miR-200c inhibited expression of p-EGFR and downstream signaling molecules PI3K 110а, p-AKT, and p-ERK, and increased the radiosensitivity of U251 (**A**), T98G (**B**), A549 (**C**), and MDA-MB-468 (**D**) cells. In contrast, miR-200c inhibition upregulated the expression of the above proteins and decreased radiation-induced cell killing. Points on the survival curves represent surviving fractions calculated from cells treated in triplicate. Each experiment was also repeated three times with similar results.

Ectopic overexpression of miR-200c increased the radiosensitivity of GBM (U251 and T98G), NSCLC (A549), and breast cancer (MDA-MB-468) cells. The sensitizer enhancement ratios (SER), calculated as the isoeffective dose to obtain 50% survival (SER_0.5_), were 1.24, 1.20, 1.05, and 1.12 for U251, T98G, A549, and MDA-MB-478 cells, respectively (Supplementary Data 1). In contrast, radiation-induced cell killing was decreased by anti-miR-200c (Figure 1A–1D).

### miR-200c overexpression induces prolongation of γH2AX focus formation and down-regulates p-DNA-PKcs

**Figure 2:**
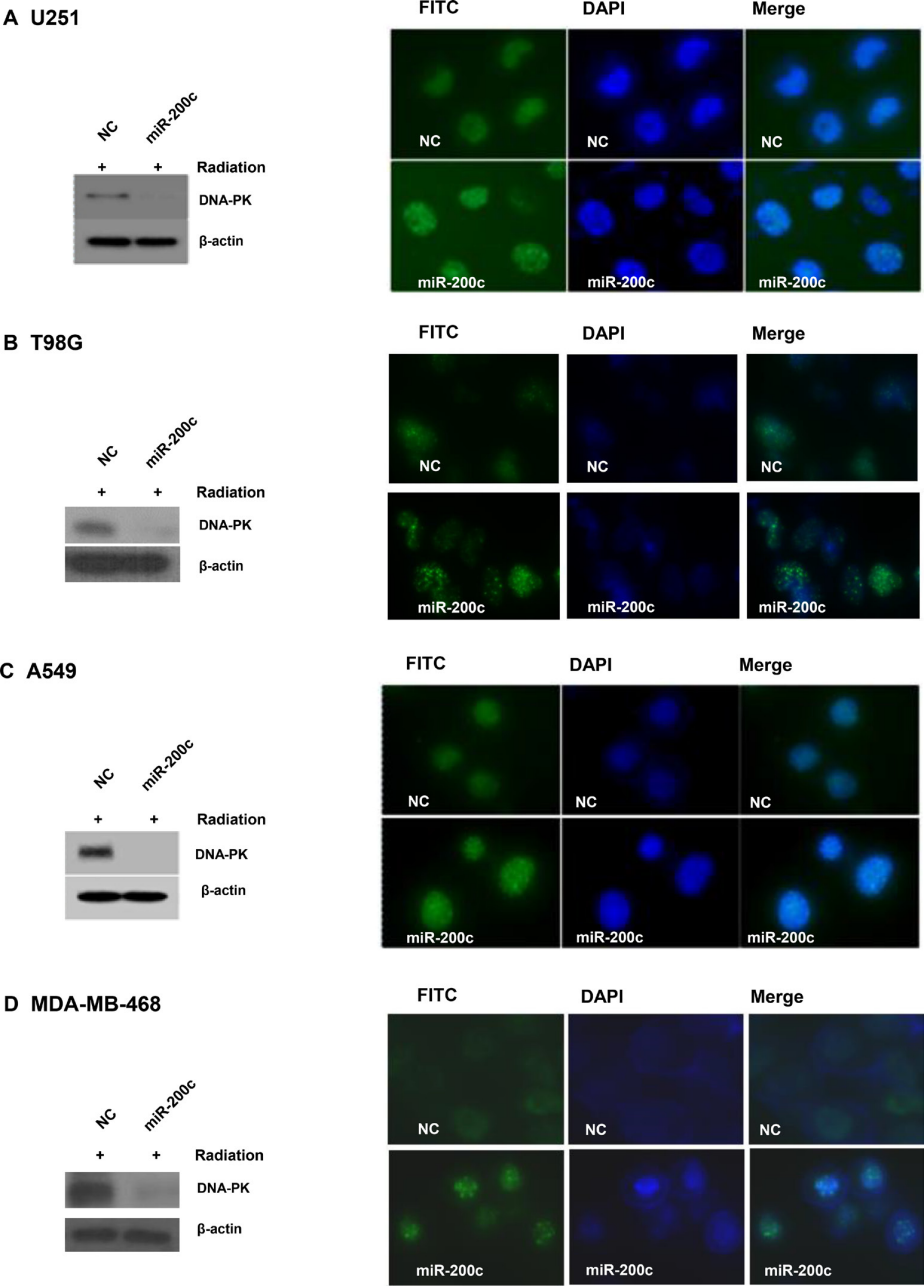
Overexpression of miR-200c led to prolonged γH2AX focus formation and p-DNA-PKcs downregulation Overexpression of miR-200c caused a marked prolongation of γH2AX focus formation at 4 hours after irradiation with 6 Gy (right panels) and was associated with discernible downregulation of p-DNA-PKcs (left panels) in U251 (**A**), T98G (**B**), A549 (**C**), and MDA-MB-468 (**D**) cells. β-actin was probed as a control.

Having demonstrated that miR-200c increased radiosensitivity in cancer cells with activated EGFR signaling, we next planned to confirm the mechanism of radiosensitization. Overexpression of miR-200c caused a marked prolongation of γH2AX focus formation 4 hours after irradiation with 6 Gy. There was no significant difference in γH2AX focus formation unless radiation was delivered (Supplementary Data 2). This was associated with a discernible downregulation of p-DNA-PKcs, which are involved in the non-homologous end joining repair process following DNA double-strand breakage (Figure 2A–2D).

### Mode of cell death: apoptosis, autophagy, and senescence

**Figure 3:**
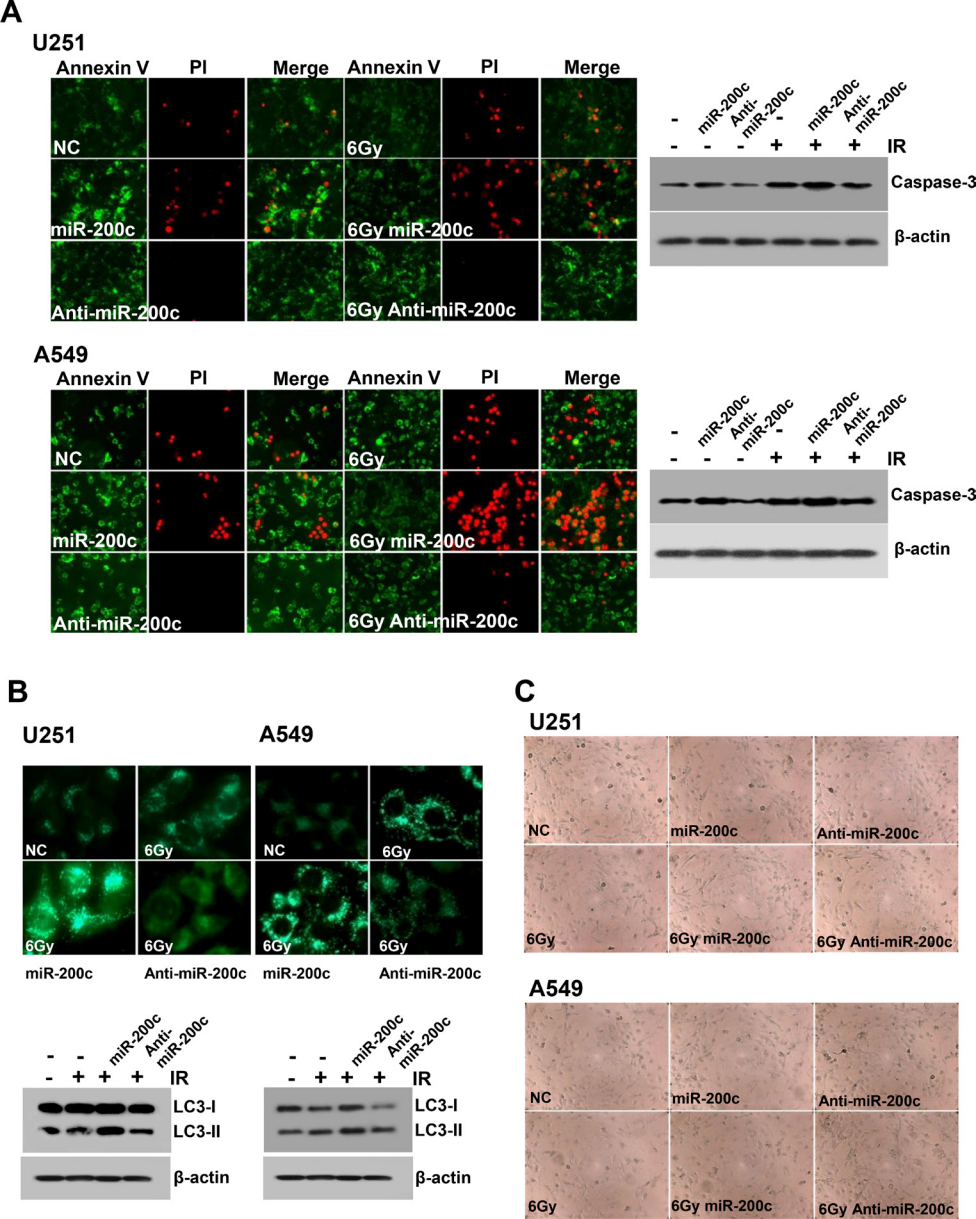
Effects of miR-200c on apoptosis, autophagy, and senescence (**A**) Annexin V/propidium iodide (PI) double staining was used to assess the degree of apoptosis or necrosis in cells with or without 6 Gy of radiation. Treatment with anti-miR-200c dramatically reduced apoptosis in U251 and A549 cells. Caspase-3 expression also correlated with apoptosis. (**B**) Treatment with both miR-200c and 6 Gy irradiation notably induced autophagy in U251 and A549 cell lines. On the other hand, anti-miR-200c treatment rendered both cell lines more resistant to autophagic cell death. Expression of the autophagosome marker LC3 was upregulated after miR-200c treatment, whereas it was reduced after anti-miR-200c treatment. (**C**) The effect of miR-200c on cellular senescence was not significant compared to normal control cells or cells treated with anti-miR-200c as determined by β-galactosidase staining.

The effect of miR-200c on apoptosis was confirmed using Annexin V/Propidium Iodide (PI) double staining [9]. Treatment of U251 and A549 cells with anti-miR-200c before irradiation significantly reduced apoptotic or necrotic cell death compared to expression of miR-200c (Figure 3A). We also examined the expression of caspase-3, a key apoptosis-triggering factor, and confirmed that caspase-3 was upregulated when U251 and A549 cells were treated with both miR-200c and radiotherapy (Figure 3A). These results showed that miR-200c and radiotherapy synergistically induced apoptotic cell death in human GBM and NSCLC cells.

Cellular stressors such as irradiation can trigger senescence signaling cascades that may promote autophagic cell death [10]. We evaluated the influence of miR-200c on the ability of U251 and A549 cells to form autophagosomes, which are associated with autophagy or autophagic cell death. Upon miR-200c overexpression, U251 and A549 cell lines showed a significantly higher degree of accumulation of acidic compartments, as demonstrated by labeling cells with LysoTracker and subsequent analysis by fluorescence microscopy. In both cell lines, treatment with miR-200c and irradiation (6 Gy) resulted in lysosomal localization of LysoTracker within 24 hours of treatment (Figure 3B). To elucidate the mechanism underlying autophagy in U251 and A549 cell lines, we investigated the effects of miR-200c and anti-miR-200c on the conversion of microtubule-associated protein light chain (LC3), an autophagosome marker. Both cell lines were positive for the unconjugated (LC3-I) and the conjugated (LC3-II) forms as determined by western blotting. However, treatment with miR-200c upregulated LC3-II expression in 24 hours (Figure 3B). The amount of LC3-II protein is associated with the number of autophagosomes [11]. According to these results, miR-200c and radiation synergistically induced autophagic cell death in GBM and NSCLC cells.

However, the effects of miR-200c on cellular senescence as determined by β-galactosidase staining indicated no significant difference from normal controls or cells treated with anti-miR-200c (Figure 3C).

### Target prediction and confirmation for miR-200c

Bioinformatics analysis predicted that EGFR had the high probability of being a miR-200c target (Supplementary Data 3). Thus, the cell lines used in the current study should be valid tools to determine this because of their activated EGFR signaling status. Western blot analysis confirmed that miR-200c overexpression decreased the expression of p-EGFR (Figure 1).

Regarding signaling pathways, we focused on pathways that were previously reported to be associated with radiosensitivity such as PI3K/Akt and ERK [12, 13]. These pathways are well known to decrease radiation-induced cell apoptosis and increase cell proliferation. Bioinformatics analysis also suggested PI3K, AKT, and ERK1/2 as putative targets of miR-200c. As presented in Figure 1, the ectopic overexpression of miR-200c led to attenuated formation of PI3K 110α, p-AKT, and p-ERK.

Angiogenesis and EMT-associated genes such as *VEGFA*, *HIF1AN*, and *CDH1*, were predicted as target genes of miR-200c. We performed western blots and validated the effect of miR-200c on VEGF, E-cadherin, and N-cadherin.

### Ectopic expression of miR-200c inhibits angiogenesis, invasion, and migration

**Figure 4:**
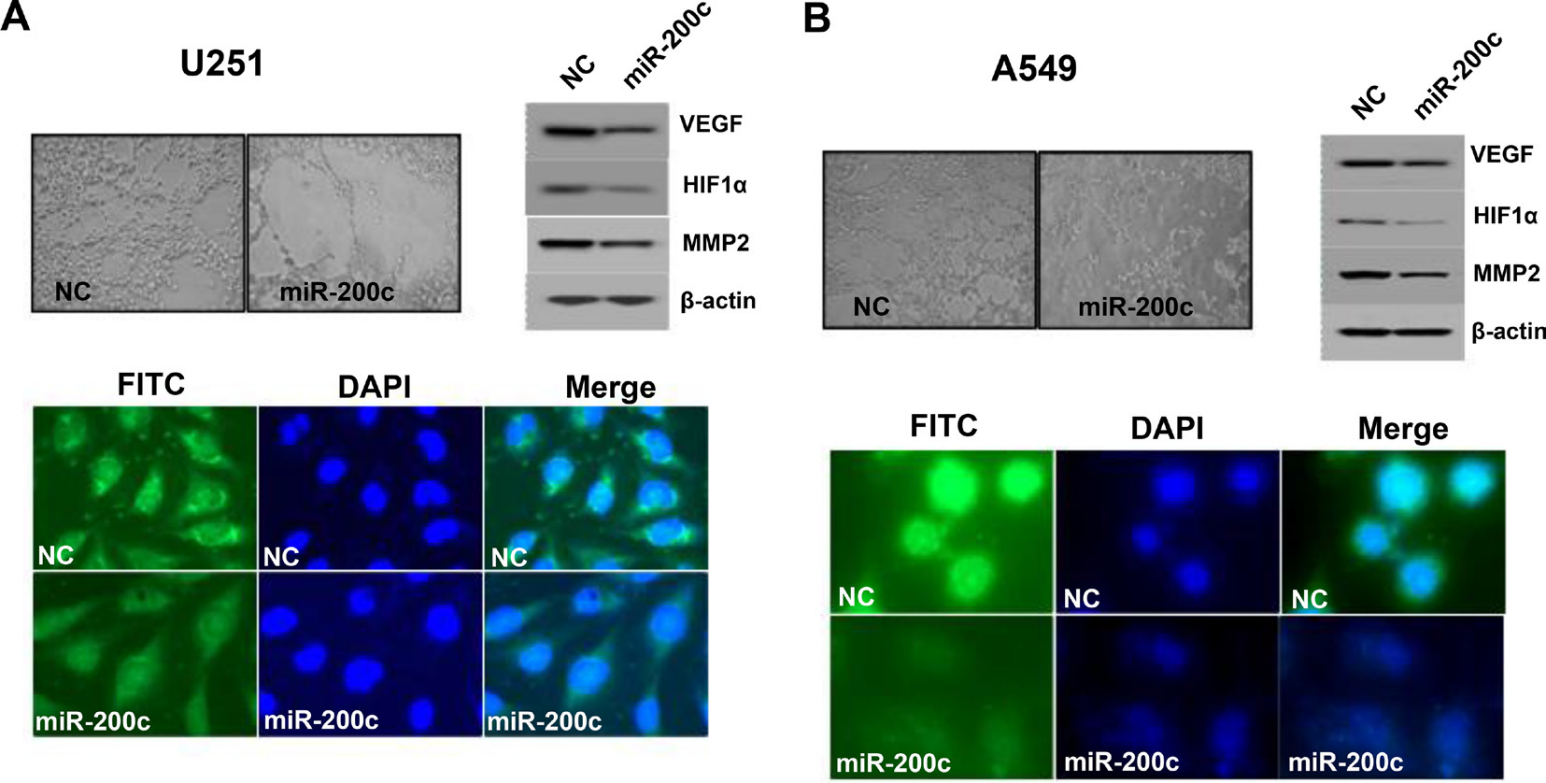
Effects of miR-200c on vascular tube formation Overexpression of miR-200c led to a discernible inhibition of vasculogenic mimicry and was associated with downregulation of VEGF, HIF-1α, and MMP2. VEGF antibody staining also confirmed the downregulation of VEGF in cell lines U251 and A549 upon treatment with miR-200c (**A** and **B**).

Overexpression of miR-200c led to discernible inhibition of vasculogenic mimicry and was associated with downregulation of VEGF, HIF-1α, and MMP2. The overexpression of VEGF in human cancer cell lines U251 and A549 was confirmed by immunocytochemistry using an anti-VEGF antibody. However, treating these cell lines with miR-200c resulted in significantly lower VEGF expression (Figure 4A and 4B).

**Figure 5:**
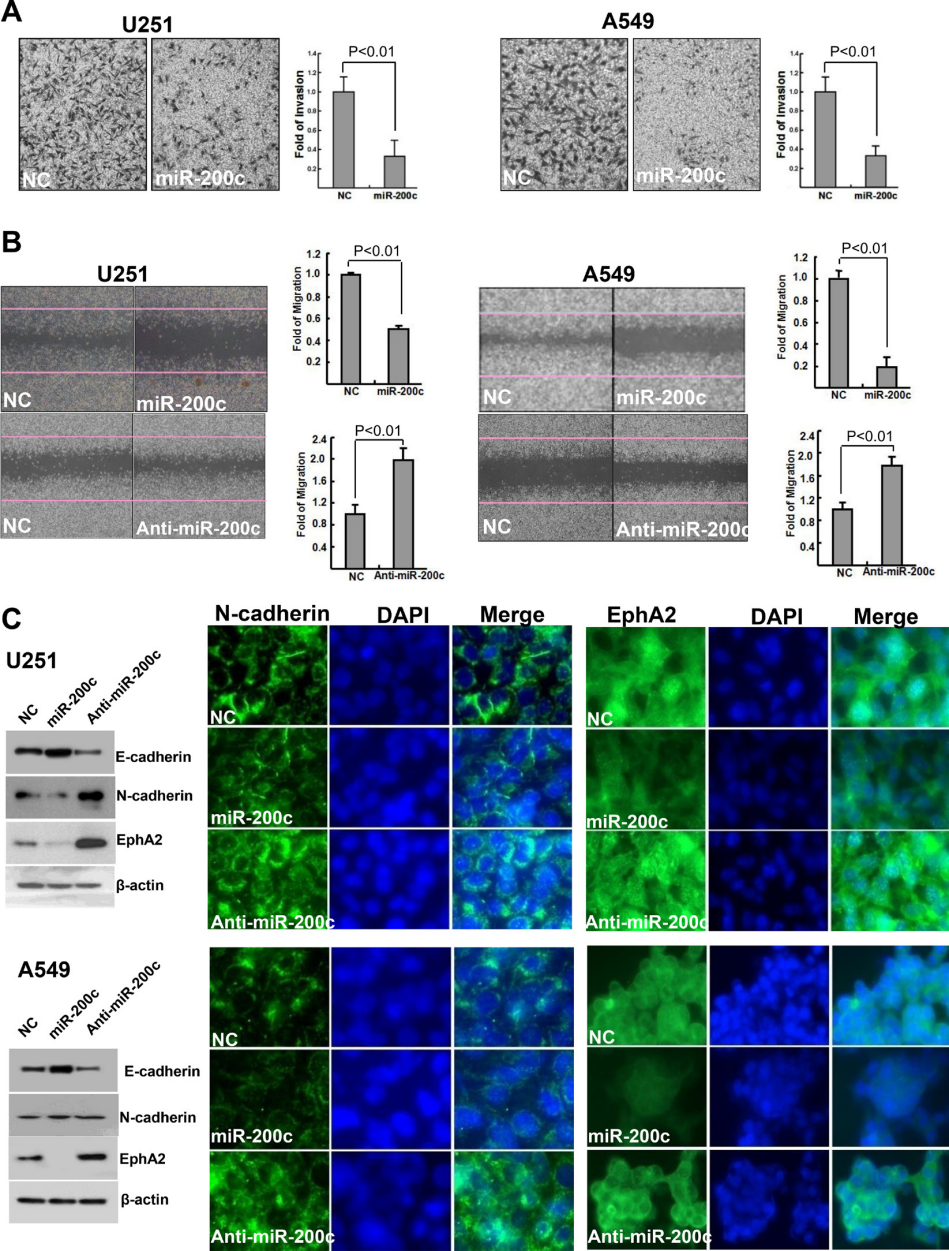
Effects of miR-200c on invasion and migration potential and E-cadherin, N-cadherin, and EphA2 expression (**A**) Overexpression of miR-200c significantly inhibited the invasion potential of U251 and A549 cells. (**B**) miR-200c significantly compromised the migration potential of U251 and A549 cells, which was restored by miR-200c inhibition. Overexpressing miR-200c increased E-cadherin expression while treatment with anti-miR-200c decreased E-cadherin expression. Anti-miR-200c also enhanced EphA2 expression whereas U251 and A549 cells incubated with anti-miR-200c exhibited a significant upregulation of EphA2 expression (**C**).

To explore the crucial role of miR200c in EMT through controlling cell migration and polarity, we evaluated invasion and migration in the panel of human cancer cells. Overexpressing miR-200c significantly inhibited the invasion potential of U251 and A549 cells (Figure 5A). miR-200c also significantly compromised migration in U251 and A549 cells as determined by wound healing assay, while a miR-200c inhibitor restored the migration potential (Figure 5B). As shown in Figure 5C, U251 and A549 cells treated with miR-200c exhibited downregulated E-cadherin and upregulated N-cadherin. Expression of EphA2 also was significantly reduced after treatment with miR-200c, whereas cells treated with anti-miR-200c had dramatically increased EphA2 expression.

### Normal cell toxicity

**Figure 6:**
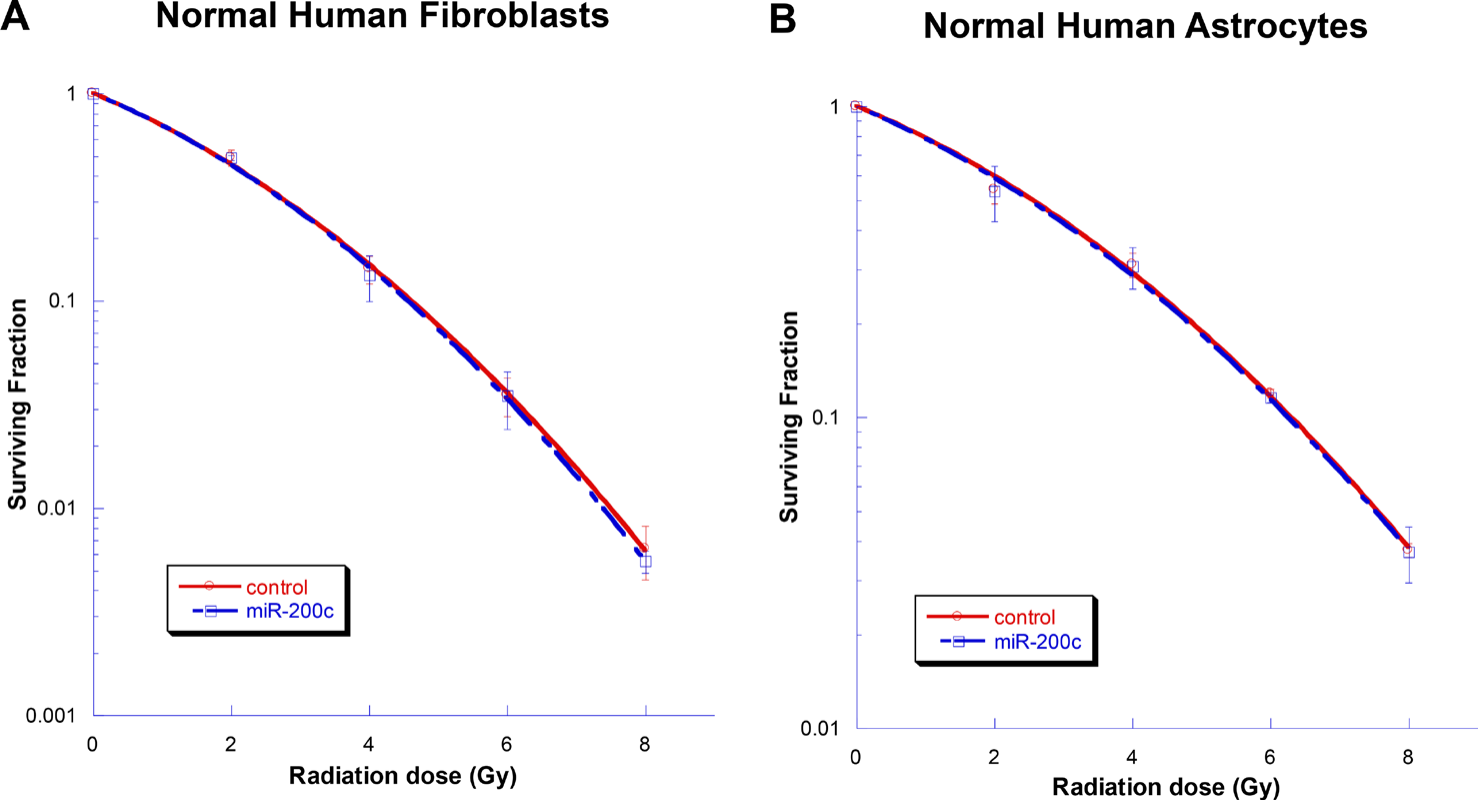
Testing toxicity of miR-200c in normal cells Ectopic overexpression of miR-200c did not alter the radiosensitivity of normal human astrocytes (**A**) or normal human fibroblasts (**B**). Points on the survival curves represent mean surviving fractions calculated from the treated cells in triplicate. Each experiment was also repeated three times with similar results.

To evaluate the toxicity of miR-200c in normal cells, a clonogenic assay was performed. The ectopic expression of miR-200c resulted in no observable cytotoxic effect on normal human fibroblasts or normal human astrocytes (Figure 6).

## DISCUSSION

miRNAs have complex interactions with mRNAs, but a few of their targets have been revealed by experiments. To overcome the limited experimental data, several miRNA target prediction algorithms have been developed. We used multiple target prediction algorithms to establish target gene candidates of miR-200c as previously reported [12], since the computational results often have inconsistencies [14]. Predicted targets were confirmed by western blotting. Our results suggested that miR-200c is an important modulator of the EGFR pro-survival cell signaling network implicated in the radiation response of human cancer cells. To the best of our knowledge, this is the first report to evaluate the regulatory role of miR-200c in response to radiation in GBM cells.

Inhibiting the PI3K/Akt signaling complex increased radiosensitivity in EGFR-activated cells by suppressing pro-survival signaling and DNA damage repair [15]. In our previous reports [12, 13], we observed that overexpressing miRNAs was associated with downregulated EGFR-associated signaling and increased radiosensitivity of human cancer cells that have activated EGFR and/or co-activation of PI3K-Akt signaling. Overexpression of miRNAs led to impaired DNA damage repair, which was associated with downregulated p-ATM or p-DNA-PK. Here we also report that overexpressing miR-200c resulted in findings indicating impaired DNA damage repair. We therefore speculated the same radiosensitization mechanism resulted from inhibiting the EGFR-associated signaling network.

The additional regulatory roles of miR-200c in important cellular signaling pathways other than PI3K-Akt or ERK have been reported. In breast cancer cells, miR-200c suppressed ubiquilin-1 expression and enhanced radiation-induced autophagy [16]. Tank-binding kinase-1 (TBK1), also a reported target of miR-200c, inhibits radiation-induced apoptosis in breast cancer and NSCLC cells [17, 18]. Enhanced chemosensitivity by miR-200c was also reported to occur through the Akt [19, 20] and JNK2/c-Jun [21] signaling pathways. Notch signaling activation was also reported to be regulated by miR-200c in human cancer cell lines [22, 23]. *Notch* genes are responsible for the expression of transmembrane receptors involved in communication between cells that are in contact. The Notch signaling network has been associated with radioresistance, and has been suggested to be a novel therapeutic target for cancer treatment [24–26].

miRNAs also affect a particular set of pro-survival cellular signals that enhance EMT, conferring cells with the ability to achieve carcinogenesis and invasiveness. Recently, a correlation between EMT and radioresistance was reported. In a gene signature study using Gene Expression Omnibus (GEO; glioma patients) and TCGA (GBM patients) datasets, samples with a radioresistant phenotype were enriched with EMT-related genes, while samples with a radiosensitive phenotype had decreased levels of EMT-related genes [27]. *In vitro*, EMT-like phenotype and E-cadherin loss induced by hypoxia contributed to radioresistance in breast cancer cells [28]. In head and neck cancer patients who had low levels of EMT-inhibiting miRNA expression pre-treatment, intrinsic radioresistance was confirmed [29].

We observed a significant loss of E-cadherin expression upon miR-200c inhibition in GBM and NSCLC cell lines. One of the first steps in the invasion of malignant cells through a weakened epithelium is loss of E-cadherin expression [30] and overexpression of N-cadherin [31]. Loss of E-cadherin in epithelial cells results in the emergence of mesenchymal characteristics during EMT [32]. We also confirmed that miR-200c inhibited the invasion and migration potential of GBM and NSCLC cell lines with activated EGFR pathways. Adam et al. [33] observed that the expression of miR-200c reversed the EMT transition in bladder cancer through the EGFR signaling pathway. Several groups also reported that the sensitivity of cancer cells to EGFR-targeted therapy is associated with E-cadherin expression and other characteristics of typical “epithelial” tumor phenotypes [32, 34–37]. These results demonstrated the critical regulatory role of miR-200c on EMT-related signaling pathways through one of the most important cell signaling pathways in cancer, EGFR signaling.

miR-200c particularly suppresses the Zinc finger E-box-binding homeobox 1 (ZEB1)/E-cadherin axis, an activator of EMT and a key promoter of metastasis [38]. Recent observations have strengthened the role of the ZEB1/miR-200c regulatory loop during EMT; on the one hand, miR-200c regulates ZEB1 expression; on the other hand, ZEB1 regulates miR-200c transcription [38, 39]. Tissue microarrays of 30 primary GBM samples reported an inverse correlation between miRNA-200c and ZEB1. GBM samples with high EGFR amplification exhibit ZEB1 upregulation and miR-200c downregulation [4]. In NSCLC cells (A549), inhibiting TBK1 attenuated radiation-induced EMT by activating glycogen synthase kinase-3β and decreasing ZEB1 expression [18]. EMT in cancer cells can lead to chemoresistance and radioresistance. In a study using doxorubicin-resistant MCF-7 breast cancer cells, loss of miRNA-200c was associated with decreased E-cadherin and PTEN expression, and increased ZEB1 and p-Akt expression, which ultimately caused chemoresistance [19].

We further confirmed another post-transcriptional role of miR-200c: inhibiting vascular formation. It is well known that HIF-1 increases vascular formation, cell survival, invasion, and radioresistance in cancer cells [40, 41]. MMP-2 and VEGF, as transcriptional targets of HIF-1, play an important role in angiogenesis of NSCLC and GBM via the PI3K/AKT signaling pathway [42, 43]. In NSCLC cells (A549), miR-200c was reported to have a radiosensitizing effect by targeting the VEGF-VEGFR2 pathway [44]. Therefore, miR-200c could be a viable target to increase radiation-induced cell killing of human tumor cells.

In this study, we found that miR-200c significantly suppressed EphA2 expression in GBM and NSCLC cells. To our knowledge, this is the first study to report a correlation between miR-200c and EphA2 in GBM and NSCLC cells. EphA2 is a member of the Eph receptor family, the largest family of tyrosine kinase receptors, which controls cell growth, migration, and differentiation. In GBM, EphA2 was overexpressed [45] and this was related with invasiveness and poor prognosis [46]. Similarly, in NSCLC, EphA2 overexpression was correlated with EGFR activation, *K-Ras* mutation, and poor prognosis [47]. Eph has been proposed as an effective therapeutic target for GBM and NSCLC due to the cross-talk that occurs between EphA2 and EGFR signaling [48–51].

In conclusion, we confirmed that miR-200c significantly enhanced the cytotoxic effect of radiotherapy in a panel of human cancer cell lines. This radiosensitizing effect was associated with attenuated EGFR-associated pro-survival signaling and impaired DNA damage repair. miR-200c also mitigated EMT-related processes such as vascular formation, invasion, and migration. Taken together, our data suggest that miR-200c is an attractive target for improving the efficacy of radiotherapy via its unique modulation of the complex regulatory network controlling cancer pro-survival signaling and EMT.

## MATERIALS AND METHODS

### Cell culture

GBM (U251 and T98G) cells, NSCLC (A549) cells, breast cancer (MDA-MB-468) cells, normal human astrocytes, and normal human fibroblasts were obtained from the American Type Culture Collection (ATCC, Manassas, VA, USA). All ATCC cell lines were authenticated by the company’s routine Cell Biology Program and were used within 6 months of receipt for this study. Cells were maintained and cultured according to standard techniques at 37°C in 5% (v/v) CO2 using the culture medium recommended by the supplier.

### miRNA transfection

Cells were transfected with pre-miR-200c (mature miR-200c sequence: 5′-CGU CUU ACC CAG CAG UGU UUG GGU GCG GUUGGG AGU CUC UAA UAC UGC CGG GUA AUG AUG GA-3′) or control pre-miRNA using siPORTNeoFX transfection reagent (Ambion, Austin, TX, USA) according to the manufacturer’s protocol.

### miRNA inhibition

To deliver anti-miR-200c (Panagene Inc., Daejeon, South Korea; Cat No.PI-1251; anti-miR-200c sequence: RRRQRRKKR-OO TTACCCGGCAGTATT) in the absence of a transfection reagent, anti-miR-200c was mixed with Opti-MEM (Invitrogen, Grand Island, NY, USA), incubated for 15 minutes at room temperature, and added directly to cells according to the manufacturer’s protocol.

### Quantitative real-time polymerase chain reaction

RT-PCR was performed using a Taqman miRNA reverse transcription kit and the Fast Real-Time PCR System (Applied Biosystems, Carlsbad, CA, USA). The fold-change of hsa-miR-200c miRNA levels was calculated and normalized to a hsa-mir-423 loading control.

### Clonogenic assay

For the clonogenic assays, identical numbers of cells were plated across the different treatment groups for each radiation dose as previously reported [52]. Cells were seeded into each well of six-well culture plates and transfected with miRNA for 18 hours. After miRNA transfection, cells were irradiated with a 4-MV X-ray from a linear accelerator (Clinac 4/100, Varian Medical Systems, Palo Alto, CA, USA) at a dose rate of 2.46 Gy/minute and incubated for colony formation for 14 to 21 days. Colonies were fixed with methanol and stained with 0.5% crystal violet; the number of colonies containing at least 50 cells was determined and the surviving fraction was calculated. Radiation-survival data were fitted to a linear-quadratic model using Kaleidagraph version 3.51 (Synergy Software, Reading, PA, USA). The SER was defined as the ratio of the isoeffective dose at a surviving fraction (SF) of 0.5 and 0.05 in the overexpression of control miRNA to the overexpression of miR-200c.

### Bioinformatics analysis

Similar to methods used in a previous study [12], several miRNA target databases were used to predict target gene candidates for miR-200c. Our analysis included miRWalk (http://zmf.umm.uni-heidelberg.de/apps/zmf/mirwalk2/index.html), MiRanda (http://www.microrna.org/microrna/home.do), miRDB (http://mirdb.org/miRDB/index.html), picTar (http://pictar.mdc-berlin.de/), PITA (http://genie.weizmann.ac.il/pubs/mir07/mir07_data.html), RNA22 (http://cm.jefferson.edu/rna22v1.0/), RNAhybrid (http://bibiserv.techfak.uni-bielefeld.de/rnahybrid/), and TargetScan (http://www.targetscan.org/). When a putative gene was predicted in four or more of the databases, we validated whether the gene was reported to be associated with radiosensitivity using the Gene (http://www.ncbi.nlm.nih.gov/gene/) and KEGG PATHWAY Databases (http://www.genome.jp/kegg/pathway.html).

### Western blot analysis

Cells were washed and suspended in lysis buffer (Cell Signaling Technology, Danvers, MA, USA). Proteins were solubilized by sonication and equal amounts of protein were separated by SDS-PAGE then electroblotted onto polyvinylidene difluoride membranes (Millipore Corp., Bedford, MA, USA). Membranes were blocked in PBS containing 0.1% Tween-20 and 5% powdered milk, and probed with the appropriate primary antibody. Primary antibodies against phosphorylated EGFR (p-EGFR; Tyr1068), p-PI3K 110а (Cell Signaling Technology), DNA-protein kinases (PKs) (Thr2609), HIF-1α, MMP2 (Abcam, Cambridge, MA, USA), p-AKT (Ser473), p-ERK1/2, VEGF, and β-actin (Santa Cruz Biotechnology, Santa Cruz, CA, USA) were used at a dilution of 1:1000. The expression level of each protein was analyzed using ImageJ (Rasband, W.S., ImageJ, U. S. National Institutes of Health, Bethesda, MD, USA, http://imagej.nih.gov/ij/, 1997–2016) program (Supplementary Data 4).

### Immunocytochemistry

Cells were grown and treated on chamber slides. At specified times after treatment with each inhibitor, cover slips were rinsed and cells were fixed in 4% paraformaldehyde then permeabilized in methanol for 20 minutes. Cells were subsequently washed and blocked in PBS containing 2% bovine serum albumin for 1 hour. Primary antibodies against γH2AX (Cell Signaling Technology), VEGF (Santa Cruz Biotechnology), EphA2 (Santa Cruz Biotechnology), LC3 (Cell Signaling Technology, Inc.), or Caspase-3 (Santa Cruz Biotechnology) were applied to the cells and incubated overnight. A secondary FITC anti-rabbit antibody (Molecular Probes, Eugene, OR, USA) was applied and incubated for 1 hour. DAPI nuclear counterstain was applied at 1 μg/mL for 5 minutes. Slides were examined on an Axio Scope.A1 Imager fluorescence microscope (Carl Zeiss, Gottingen, Germany. Images were captured using an AxioCam MRc5 and acquisition software AxioVision v.4.4 (Carl Zeiss).

### Annexin V/propidium iodide double-staining

Apoptosis was examined using Annexin V/propidium iodide (PI) double staining. Cells were seeded onto an 8-well chamber slide, doubled stained with Annexin V and propidium iodide according to the manufacturer’s instructions, and then subjected to the analyzer (BD, Franklin Lakes, NJ, USA).

### Cell labeling with lysotracker

To detect autophagy, cells were incubated with 1 µM LysoTracker (Molecular Probes, Eugene, OR, USA) for 10 seconds during irradiation according to the manufacturer’s instructions. After incubation, cells were washed with PBS and analyzed by fluorescence microscopy.

### β-galactosidase staining for senescence

Cellular senescence was evaluated using a β-Galactosidase Staining Kit (Cell Signaling Technology). Medium was removed and cells were washed with PBS. The β-galactosidase staining solution (1 µL) was added to each 35 mm well and cells were incubated at 37°C overnight according to the manufacturer’s instructions.

### Wound healing assay

Cells (5 × 10^6^ cells/well) at 48 hours post-transfection were counted and seeded into six-well tissue culture plates (SonicSeal Slide; Nalge Nunc, Rochester, NY, USA) and grown until 90% confluence. A vertical or horizontal wound was created using a 1000 mL pipette tip. The wounded cells were rinsed three times with 1× PBS to remove all cellular debris. Serum-free medium was added and the cells were allowed to migrate for 48 hours. Images were microscopically captured at designated times to assess wound closure. The inhibition effectiveness was estimated by the relative distance of wound closure.

### Modified Boyden chamber assay

Cell invasion was measured using a Transwell system (Corning, Rochester, NY, USA), which allows cells to migrate through 8 µm pores in polycarbonate membranes. The membranes were coated with a 10 µg/well gelatin solution in starvation medium and subsequently dried. Inserts containing cells were placed into 24-well plates (Corning) in starvation medium. Cells were trypsinized, washed, and resuspended (5 × 10^6^ cells/mL) in starvation medium. Cells (10^4^) were added to the upper chamber. The lower chamber was filled with 500 µL medium. After 24 hours, the surface of the upper membrane was swabbed with a cotton-tipped applicator to remove remaining cells. Inserts were fixed in methanol for 10 minutes and stained with 1% crystal violet for 2 hours. Invasion rate was estimated microscopically from the number of cells that migrated into the lower chamber.

### Vasculogenic mimicry formation assay

Vasculogenic mimicry formation assay was performed using a commercialized Matrigel assay kit (BD Biosciences). ECM Matrigel (200 μL) was added to 48-well tissue culture plates and then incubated at 37°C for 2 hours. Cells were transfected with pre-miR-200c or control 1 day before seeding onto coated plates. After growth for 24 hours on the plate, vasculogenic mimicry formation was assessed using an inverted microscope.

### Statistical analysis

Statistical analysis was performed by Student’s *t*-test using IBM SPSS ver. 20 (IBM Corp., Armonk, NY). *p*-values less than 0.05 were considered statistically significant.

## SUPPLEMENTARY MATERIALS FIGURES




