# Supplementary material for: MicroRNA-200c increases radiosensitivity of human cancer cells with activated EGFR-associated signaling
Source: Oncotarget. 2017 Jul 3;8(39):65457–68. doi: 10.18632/oncotarget.18924 (PMC5630345; doi:10.18632/oncotarget.18924)
Supplement: Supplementary file 1 [file oncotarget-08-65457-s001.pdf]

MicroRNA-200c increases radiosensitivity of human cancer cells with activated EGFR-associated signaling

SUPPLEMENTARY MATERIALS

miR-200c

SER / Cell	$SF_{0.5}$	$SF_{0.05}$
U251	1.24	1.25
T98G	1.05	1.11
A549	1.20	1.18
MDA-MB-468	1.12	1.10

Anti-miR-200c

SER / Cell	$SF_{0.5}$	$SF_{0.05}$
U251	1.00	0.92
T98G	0.86	0.88
A549	1.00	0.88
MDA-MB-468	0.94	0.88

Supplementary Data 1: Sensitizer enhancement ratio (SER).

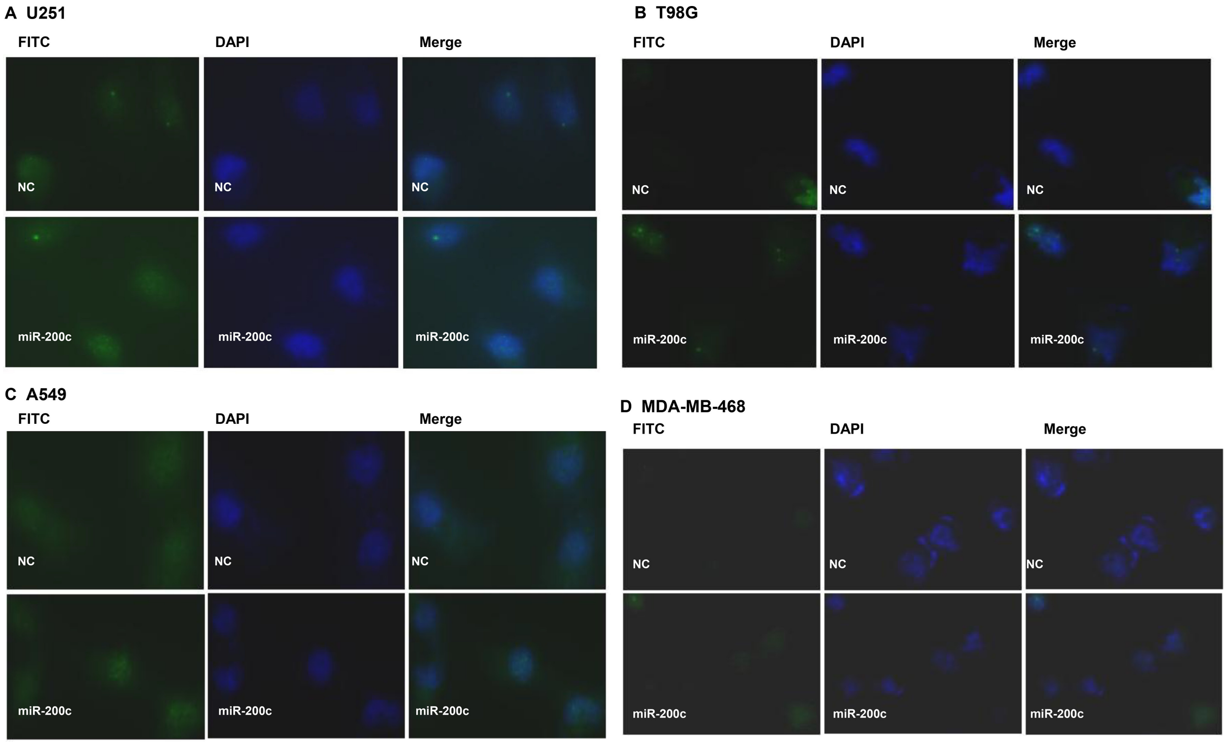

Supplementary Data 2: Overexpression of miR-200c had no significant difference in γH2AX focus formation unless radiation was delivered.

miRNA	Gene (Protein)	miRWalk	miRanda	miRDB	Pictar2	PITA	RNA22	RNAhybrid	Targetscan	SUM
hsa-miR-200c	*EGFR* (EGFR)	1	1	0	0	0	1	1	1	5
hsa-miR-200c	*PIK3CB* (PI3K)	1	1	0	1	0	0	1	1	5
hsa-miR-200c	*AKT2* (AKT)	0	1	0	0	1	1	1	1	5
hsa-miR-200c	*MAPK1* (ERK1/2)	1	0	0	0	1	1	1	0	4
hsa-miR-200c	*VEGFA* (VEGF)	1	1	0	1	0	0	1	1	5
hsa-miR-200c	*HIF1AN* (HIF-1α inhibitor)	1	1	1	0	1	0	1	1	6
hsa-miR-200c	*CDH1* (E-cadherin)	1	0	0	0	0	1	1	1	4

Supplementary Data 3: Target gene prediction using miRNA target databases.

Supplementary Data 4: See Supplementary_Data_4
